# Dietary behaviours during COVID-19 among households at risk for food insecurity

**DOI:** 10.1017/jns.2023.36

**Published:** 2023-05-02

**Authors:** Maha Almohamad, Allison Marshall, Jayna Markand Dave, Ru-Jye Chuang, Christine Markham, Shreela Sharma

**Affiliations:** 1 Department of Epidemiology, Human Genetics and Environmental Sciences, The University of Texas Health Science Center at Houston (UTHealth) School of Public Health, 1200 Pressler Street, Houston, TX 77030, USA; 2 Michael & Susan Dell Center for Healthy Living, The University of Texas Health Science Center at Houston (UTHealth) School of Public Health in Austin, 1616 Guadalupe Street, Austin, TX 78701, USA; 3 USDA/ARS Children's Nutrition Research Center, Baylor College of Medicine, 1100 Bates Ave., Houston, TX 77030, USA; 4 Department of Health Promotion and Behavioral Sciences, The University of Texas Health Science Center at Houston (UTHealth) School of Public Health, 7000 Fannin Street, Houston, TX 77030, USA

**Keywords:** Behavioural changes, COVID-19, Food insecurity, Healthy eating, Low income

## Abstract

The objective of the present study was to examine associations between variables of COVID-19-related concerns and changes in fruit and vegetable (FV) consumption among a sample of participants from the Brighter Bites program at risk for food insecurity. Cross-sectional data were collected during April–June 2020 using a rapid-response survey to understand social needs, COVID-19-related concerns and diet-related behaviours among families with children participating in Brighter Bites (*n* 1777) in the 2019–2020 school year at risk for food insecurity, within the surrounding Houston, Dallas, Austin, Texas area; Southwest Florida; Washington, D.C., United States. Of the 1777 respondents, 92 % of households reported being at risk for food insecurity. Among those from food insecure households, the majority were of Hispanic/Mexican-American/Latino (84⋅1 %) ethnic background, predominantly from Houston, Texas (71⋅4 %). During the pandemic, among individuals from food insecure households, 41 % (*n* 672) reported a decrease in FV intake, 32 % (*n* 527) reported an increase in FV intake, and 27 % (*n* 439) reported no change in FV intake. Those who reported concerns about financial stability had a 40 % greater risk of decreased FV intake compared to those not concerned about financial stability (RR 1⋅4; 95 % CI 1⋅0, 2⋅0; *P* = 0⋅03). The present study adds to this current body of sparse literature on how the initial phase of the pandemic impacted FV consumption behaviours among food insecure households with children. Effective interventions are needed to diminish the negative impact of COVID-19 on the population's health.

## Introduction

In March 2020, the World Health Organization (WHO) declared the coronavirus disease (COVID-19) as a global pandemic^(1)^. Subsequently, the United States (US) declared a national state of emergency, with state and local stay-at-home orders, closing schools and businesses. Economic implications including unemployment, food insecurity, financial instability and access to healthcare services worsened, deeply impacting low-income populations^(2)^. Preventive measures became unavoidable, influencing lifestyle-related habits, food choices and diet. Through the monitoring of the pandemic's effect food spending, prices and sufficiency, the USDA (US Department of Agriculture), Economic Research Service (ERS) found expenditures at restaurants and other eating-out establishments fell from $79 billion in February 2020 to $42 billion in April 2020^(3)^. Furthermore, COVID-19 illnesses led to a reduction of farm workers leading to decreased production of fresh vegetables and crops^(3)^. Prior to the COVID-19 pandemic, in 2019, 10⋅5 % (13⋅7 million) of US households were food insecure, 13⋅6 % of which included children^(4)^. As a result of the pandemic and the subsequent financial crisis, food insecurity rates increased. At the time, population experts had projected more than 42 million individuals may experience food insecurity, with approximately 13 million being children in 2021^(4)^. Food insecurity has been recognised as a critical social determinant of health with important links to health and behavioural outcomes^(5)^.

Food insecurity is defined as the lack of or uncertain availability and limited access to nutritionally adequate food acquired in socially acceptable ways^(6)^. As determined by the USDA (US Department of Agriculture), food insecurity was associated with reduced food intake and disrupted eating patterns among households with children due to lost school meals support during the pandemic with shut down of schools^(4,7)^. Furthermore, food insecurity has been associated with consumption of lower quality food, poor nutrition and changes in dietary habits, specifically reduced fruit and vegetable (FV) consumption^(8)^. Additionally, purchasing packaged foods with longer shelf lives, rather than fresh food, led to lower consumption of the recommended portions of FV^(9)^. A recent cohort study showed disruption of dietary behaviours during the COVID-19 lockdown^(10)^. Results of the present study indicated increased weight gain due to increased snacking and sedentary activity, and decreased fresh produce consumption^(10)^. The study also examined shopping behaviours with 27 % of individuals reported buying less fresh produce and 26 % reported difficulty in accessing fresh produce^(10)^.

Brighter Bites is a school-based nutrition and health promotion programme serving families with children in schools with 75 % of the student population eligible for free or reduced-price lunches. The programme uses a co-op model to distribute fresh FV to low-income families in elementary schools and early childcare centres in conjunction with nutrition education, recipes and recipe demonstrations^(11)^. Brighter Bites programming had been implemented during the school year in two 8-week sessions in the fall and spring, with additional summer programming for up to 8 weeks^(11)^. In March 2020, due to COVID-19-related school closures, Brighter Bites programming came to a halt.

Limited studies have assessed the impact of COVID-19-related environmental shifts on FV intake among food insecure families with children within the US. To understand the impact of the early phase of the pandemic, in April–June 2020, Brighter Bites conducted a rapid assessment of dietary behaviours, social determinants of health and COVID-19 concerns among participating families experiencing food insecurity. The purpose of the present study was to examine the cross-sectional relationship between both COVID-19-related concerns as well as FV shopping behaviours with self-reported changes in FV consumption among a sample of food insecure families with children enrolled in the Brighter Bites program.

## Methods

### Design

Cross-sectional data were collected during April–June 2020 as part of a rapid-response survey to understand social needs, COVID-19-related concerns and diet-related behaviours among families with children participating in Brighter Bites in the 2019–2020 school year at risk for food insecurity. Ethical approval for this study was obtained from the Institutional Review Board at the University of Texas Health Science Center (UTHSC) at Houston (IRB# HSC-SPH-15-0752).

### Respondents and recruitments

The rapid-response survey screener was electronically distributed in both English and Spanish versions via Formsite (Vroman Systems, Inc.) to participants who provided a telephone number and consent during enrolment in Brighter Bites. A total of 16 673 participating Brighter Bites families in Texas (4⋅0 % in Austin, 56⋅2 % in Houston and 25⋅7 % in Dallas), Southwest Florida (6⋅1 %) and Washington, D.C. (8⋅1 %) received a texted link from programme staff members. The survey was voluntarily completed by a parent or adult caregiver in the family and open for 3 months (April–June 2020) with a response rate of 10⋅7 % (1777 of 16 673). The survey is typically distributed at this time, at the end of the school year to assess the effect of the Brighter Bites program within the past Spring semester. This specific survey was distributed to report changes in activity due to the pandemic along with school closures. Response rates by region were 4⋅8 % in Austin, 12⋅5 % in Houston and 5⋅2 % in Dallas, Texas; 12⋅3 % in Southwest Florida and 6⋅9 % in Washington, D.C. Response rates may have been low due to school closures and changes in people's lives due to the pandemic, even though researchers followed up with a reminder link to complete the survey on a weekly basis for 3 months. The proposed response rate was expected to have been 70 % or higher, similar to previous assessment years^(12)^. Furthermore, Brighter Bites collected, de-identified and shared completed data with UTHSC for analysis as part of a data-sharing agreement.

### Data collection and measures

The self-reported 30-item survey, pilot tested within the research team prior to full scale administration, took about 10 min to complete, and was administered electronically with accessibility on smartphones or computers. The survey was used to rapidly assess socio-demographic data, dietary and FV shopping behaviours, COVID-19-related concerns and social determinants of health described below.

#### Socio-demographic data

Respondent socio-demographic data included sex, age, relationship to child, respondent and child race/ethnicity, employment status, education level, and total number of adults and children in household. Respondents’ enrolment in government assistance programmes (Special Supplemental Nutrition Program for Women, Infants, and Children (WIC), Supplemental Nutrition Assistance Program (SNAP), Double Dollars, and/or free or reduced-price National School Lunch Program (NSLP)) was also assessed. Participation in food assistance programmes was coded as participating in none, one, or two or more programmes.

#### Outcome: parental FV intake

As a result of the pandemic, parent's FV intake was measured using a created 3-item self-reported block screener based on a previously validated self-reported FV block screener^(13)^. We were interested in knowing whether the overall frequency of FV intake had changed or stayed the same, rather than knowing changes in portion sizes or specific food items. The frequency of FV intake was assessed using the following question ‘Due to coronavirus, has your consumption of fruits and vegetables: increased, decreased or stayed the same?’ We were able to compare responses to this survey in Spring 2020 to the past survey in Fall 2019 before the peak of the pandemic. Moreover, questions within this survey are specifically geared to assess changes due to COVID-19 by tweaking the already validated questions to include changes due to COVID-19 and distributing these surveys during Spring 2020.

#### Exposure: FV shopping behaviour

FV shopping frequency and behaviour were characterised using items adapted from the National Cancer Institute's 2007 Food Attitudes and Behavior Survey^(14)^. Both frequency (times/month or times/week) and type of store for FV purchase were assessed using the following questions and response options: ‘Due to coronavirus, currently how often do you buy or get fruits and vegetables and other groceries for the family from a large grocery store or super market?’ Five response options ranging from: ‘never’ to ‘2+ times per week’. ‘Due to coronavirus, currently how often do you buy or get fruits and vegetables and other groceries for the family from these locations?’ Response options: ‘A small local store or corner store (usually locally owned and do not sell gas), or a convenience store (such as 7–11 or mini market usually sell gas)’, ‘farmer's market/food co-op/farm stand’ and ‘food bank/food pantry, or other food distributions’. Survey items were modified by adding ‘Due to coronavirus’ to reflect the behavioural changes due to COVID-19 among an already food insecure population. Questions do not reflect shopping behaviours for items they need due to COVID-19, but rather their shopping behaviour for FV specifically during the pandemic.

Multiple responses could be selected for these questions. Shopping behaviour was categorised as the total number of times per month or per week an individual shopped for FV at a certain location. For every one time per month, frequency was assessed as a continuous variable and coded 0 (never), 0⋅5 (less than once a month), 1⋅5 (1–2 times per month), 4 (1 time per week) and 8 (2+ times per week). We tweaked the questionnaire and asked that the respondents answer these questions with the changes of their behaviours being due to the pandemic with no other modifications made to the specific questions. Moreover, we were able to compare responses during the pandemic in Spring 2020 to responses pre-pandemic in Fall 2019.

#### Exposure: variables for COVID-19-related concerns

The items assessing COVID-19-related concerns were adapted from previously validated survey instruments^(15)^: ‘Due to coronavirus, are you concerned about any of the following in regards to you and your family? (Check all that apply)’ Multiple response options could be selected from: ‘Financial stability’, ‘My employment status will change in the near future’, ‘Availability of food’ and ‘Affordability of food’.

### Confounders

#### Eating out frequency

As a result of the pandemic, the family's frequency of eating food from a restaurant was measured using a created 3-item self-reported block screener, by previously validated items for home mealtime environment^(12,16)^. We were interested in knowing whether the overall frequency of eating out had changed or stayed the same. The frequency of eating out was assessed using the following question, ‘During the past 7 days, how many times did your family: Eat food from any type of restaurant? This includes restaurants such as fast food, sit-down restaurants, buffet restaurants, taco shops, donut shops and pizza places’. Five response options ranged from ‘never’ to ‘7+ times per week’.

#### Food insecurity

The 2-item Hunger Vital Sign Screener assessed household food security status during the COVID-19 pandemic^(17)^. We added the phrase ‘due to coronavirus’ to each of the 2-item screener to assess the risk of food insecurity that is due to the pandemic, rather than general risk of food insecurity. The following items were worded as such: ‘Due to coronavirus: You worried whether your food would run out before you got money to buy more’ and ‘The food you bought just didn't last and you didn't have money to get more’^(17)^. Response options for both items included ‘Often True’, ‘Sometimes True’ or ‘Never True’. Household respondents who responded ‘Often True’ or ‘Sometimes True’ to either food security question was categorised as at risk for food insecurity. Households were categorised as food secure if respondents responded ‘Never True’ to both questions.

### Statistical analysis

At baseline, the sample included 1777 adults participating in Brighter Bites, 92⋅2 % of whom reported experiencing food insecurity. Therefore, we only included those who were at risk for food insecurity in the analysis for this current study (*n* 1639). Since those who were food secure represented less than 10 % of the population, we did not include them in this analysis as a potential comparison group. All analyses were conducted using Stata version 16.0 (StataCorp LLC). Descriptive statistics and bivariate analyses, including Chi-square, examined differences in respondent characteristics. Means, standard deviations and frequency distributions were computed. Frequency distributions were stratified by a categorical FV intake. During this acute phase of the pandemic, multinomial logistic regression modelling was used to determine cross-sectional associations between FV shopping behaviours and changes in FV intake using Beta coefficients. Furthermore, multinomial logistic regression modelling was used to determine cross-sectional associations between variables for COVID-19-related concerns and changes in FV intake using relative risk ratios. The main dependent variable assessed was a self-reported change in FV intake due to the COVID-19 pandemic. The independent variables were (a) COVID-19-related concerns such as financial stability, change of employment status, food availability and food affordability, and (b) FV shopping behaviour. Confounding variables were chosen *a priori* using a directed acyclic graph and computing the measure of association both before and after adjusting^(18)^. Moreover, confounding was present and included in the model if the difference between the two measures of association was at least 10 %^(19)^. Models were adjusted for the following covariates: race/ethnicity, region, total number of adults and children in household, frequency of eating out at restaurants, food assistance criteria and FV shopping behaviours in only those who were food insecure based on the level of significance. The significance level used was *P* < 0⋅05.

## Results

### Descriptive statistics

Table 1 outlines socio-demographic characteristics of food insecure households (*n* 1639). Of these food insecure households, mean respondent age was 37 years old (±7⋅2); respondents were mostly Hispanic (84⋅1 %), predominantly located in Houston, Texas (71⋅4 %). Seventy-three percent reported their children utilised NSLP's free or reduced-price meals, 31 % received SNAP benefits and 25 % received WIC. Furthermore, 16 % of respondents reported not utilising any food assistance programmes, 48 % used only one programme and 37 % used two or more programmes. Food insecurity in households was positively associated with food assistance programme participation (*P* < 0⋅001).

**Table 1.** Descriptive table of survey/respondent characteristics with food insecurity bivariate relationship between food insecurity and covariates

Variable	Food insecure *n* 1639	*P*-value
Age *n* (in years, Mean (sd))	1483 (36⋅7 (7⋅2))	0⋅09
Race *n* (%)		
White, Caucasian, Anglo	55 (3⋅4 %)	<0⋅001*
Black, African American	137 (8⋅4 %)	
Mexican-American, Latino, Hispanic	1369 (84⋅1 %)	
Other	66 (4⋅1 %)	
Region (Column %)		
Houston	1170 (71⋅4 %)	0⋅47
Dallas	221 (13⋅5 %)	
Austin	32 (2⋅0 %)	
SW Florida	124 (8⋅0 %)	
D.C.	92 (5⋅6 %)	
Total number of people living in home *n* (Mean (sd))	1639 (4⋅8 (1⋅9))	0⋅04*
Total number of adults living in home *n* (Mean (sd))	1509 (2⋅3 (0⋅9))	0⋅002*
Total number of children living in home *n* (Mean (sd))	1534 (2⋅7 (1⋅2))	0⋅003*
‘Due to coronavirus, are you concerned about any of the following in regards to you and your family? (Check all that apply)’ *n* (% yes)		
Financial stability	1277 (77⋅9 %)	<0⋅001*
My employment status will change in the near future	674 (41⋅1 %)	<0⋅001*
Availability of food	1146 (69⋅9 %)	<0⋅001*
Affordability of food	871 (53⋅1 %)	<0⋅001*
Family eat food from restaurant		
Never	1020 (62⋅3 %)	<0⋅001*
1–2 times/week	573 (35⋅0 %)	
3–4 times/week	35 (2⋅1 %)	
5–6 times/week	6 (0⋅4 %)	
7+ times/week	4 (0⋅2 %)	
Fruit and vegetable intake		
Stay the same	439 (26⋅8 %)	<0⋅001*
Decreased	672 (41⋅0 %)	
Increased	527 (32⋅2 %)	
Food assistance programmes *n* (% yes)		
WIC	415 (25⋅4 %)	0⋅02*
SNAP	513 (31⋅5 %)	0⋅02*
Double Dollars	21 (1⋅3 %)	0⋅87
Free/reduced-price meals	1200 (73⋅4 %)	<0⋅001*
Food assistance criterion		
None	255 (15⋅6 %)	<0⋅001*
One programme	786 (48⋅0 %)	
Two or more programmes	598 (36⋅5 %)	
FV shopping behaviour	Food insecurity *n* 1639	*P*-value
Due to coronavirus, currently how often do you buy or get fruits and vegetables and other groceries for the family from these locations?		
Large grocery or supermarket		
Never/less than once a month	256 (15⋅6 %)	0⋅04*
1–2 times/month	590 (36⋅0 %)	
1 time/week	560 (34⋅2 %)	
2+ times/week	233 (14⋅2 %)	
Small local store or corner store		
Never/less than once a month	1043 (63⋅7 %)	0⋅05*
1–2 times/month	270 (16⋅5 %)	
1 time/week	249 (15⋅2 %)	
2+ times/week	75 (4⋅6 %)	
Farmers market or food co-op		
Never/less than once a month	1463 (89⋅5 %)	0⋅54
1–2 times/month	77 (4⋅7 %)	
1 time/week	61 (3⋅7 %)	
2+ times per week	33 (2⋅0 %)	
Food bank or food pantry		
Never/less than once a month	1079 (66⋅0 %)	0⋅52
1–2 times/month	289 (17⋅7 %)	
1 time/week	206 (13⋅0 %)	
2+ times/week	62 (3⋅8 %)	

WIC, Special Supplemental Nutrition Program for Women, Infants, and Children; SNAP, Supplemental Nutrition Assistance Program; sd, standard deviation.

* *P* < 0⋅05; *P* < 0⋅01; *P* < 0⋅001; or Significance based on 95 % CI.

Statistical test used: Chi-square.

Among individuals from our final sample, 41 % (*n* 672) reported decreased FV intake during the pandemic, 32 % (*n* 527) reported increased intake and 27 % (*n* 439) reported no change in intake. Moreover, 62 % of respondents reported never eating out at a restaurant during the pandemic, while 38 % reported eating food from a restaurant at least once a week. During the time of the pandemic, 16 % (*n* 256) reported either never shopping or shopping less than once a month at large grocery stores/supermarket. Among those who shopped at large grocery stores/supermarket, most respondents shopped either 1–2 times per month (36 %) or 1 time per week (34 %). The majority of respondents (64 %; *n* 1043) reported frequency of shopping as never or less than once a month at a small local store/corner store for FV. A smaller percentage of respondents (34 %) reported shopping less than once a month at a food bank/food pantry.

Respondents reported a high prevalence of concerns regarding financial stability (78 %), changes in employment status (41 %), food availability (70 %) and food affordability (53 %).

### Relationship between FV shopping behaviours and changes in FV consumption among respondents from food insecure households

Among respondents whose FV intake decreased, shopping frequency at large grocery stores/supermarket was 2⋅3 [±2⋅0] times per month. However, among individuals whose FV intake stayed the same, frequency of shopping at large grocery stores/supermarket was 3⋅2 [±2⋅2] times per month. There was a positive association between frequency of FV shopping and changes in FV intake (Adj*β* = −0⋅2, CI −0⋅3, −0⋅1; *P* < 0⋅001). Similarly, among respondents who reported increased FV intake during this time, frequency of shopping at a large grocery store/supermarket was 4⋅2 [±2⋅6] times per month (*β* = 0⋅2, CI 0⋅1, 0⋅2; *P* < 0⋅001).

The frequency of shopping at small local stores/corner stores was 1⋅1 [±1⋅7] times per month among those whose FV intake decreased. In contrast, among individuals whose FV intake stayed the same, the frequency of shopping at small local stores/corner stores was 1⋅3 [±2⋅1] times per month (*β* = −0⋅1, CI −0⋅2, −0⋅0; *P* = 0⋅006) (Table 2). There was no significant relationship between increased FV intake and FV shopping behaviour at small local stores/corner stores.

**Table 2.** Relationship between changes in FV intake and FV shopping behaviours, and concerns regarding various social determinants of health among those who were at risk for food insecurity (*N* 1639)

Variable name	Changes in fruit and vegetable intake					
	Stay the same 439 (26⋅80 %)		Decreased 672 (41⋅03 %)		Increased 527 (32⋅17 %)	
	*N* (Mean (sd))	Beta (95 % CI) *P*-value (REF)	*N* (Mean (sd))	Beta (95 % CI) *P*-value	*N* (Mean (sd))	Beta (95 % CI) *P*-value
FV shopping behaviours						
Large grocery or supermarket	504 (3⋅2 (2⋅2))	(REF)	701 (2⋅3 (2⋅0))	−0⋅2 (−0⋅3, −0⋅1) <0⋅001*	571 (4⋅2 (2⋅6))	0⋅2 (0⋅1, 0⋅2) <0⋅001*
Small local store or corner store	504 (1⋅3 (2⋅1))	(REF)	700 (1⋅1 (1⋅7))	−0⋅1 (−0⋅2, 0⋅0) 0⋅006*	571 (1⋅5 (2⋅3))	0⋅0 (−0⋅0, 0⋅1) 0⋅20
Farmers market or food co-op	503 (0⋅4 (1⋅2))	(REF)	698 (0⋅4 (1⋅1))	−0⋅1 (−0⋅2, 0⋅0) 0⋅19	571 (0⋅6 (1⋅6))	0⋅1 (−0⋅1, 0⋅2) 0⋅07
Food bank or food pantry	504 (1⋅1 (1⋅8))	(REF)	699 (1⋅0 (1⋅7))	−0⋅1 (−0⋅1, 0⋅0) 0⋅21	571 (1⋅4 (2⋅1))	0⋅1 (0⋅0, 0⋅1) 0⋅05*
COVID-19-related concerns	*N* (% yes)	RR (95 % CI) *P*-value (REF)	*N* (% yes)	RR (95 % CI) *P*-value	*N* (% yes)	RR (95 % CI) *P*-value
Financial stability	326 (74⋅3 %)	(REF)	535 (79⋅6 %)	1⋅4 (1⋅0, 2⋅0) 0⋅03*	415 (78⋅8 %)	1⋅3 (0⋅9, 1⋅8) 0⋅126
My employment status will change in the near future	151 (34⋅4 %)	(REF)	273 (40⋅6 %)	1⋅3 (1⋅0, 1⋅7) 0⋅064	250 (47⋅4 %)	1⋅7 (1⋅3, 2⋅3) <0⋅001*
Availability of food	276 (62⋅9 %)	(REF)	497 (74⋅0 %)	1⋅7 (1⋅3, 2⋅2) <0⋅001*	373 (70⋅8 %)	1⋅5 (1⋅1, 2⋅0) 0⋅01*
Affordability of food	204 (46⋅5 %)	(REF)	373 (55⋅5 %)	1⋅5 (1⋅1, 2⋅0) 0⋅003*	294 (55⋅8 %)	1⋅4 (1⋅1, 1⋅8) 0⋅02*

RR, relative risk ratio; CI, confidence interval; sd, standard deviation; REF, Reference.

* *P* < 0⋅05; *P* < 0⋅01; *P* < 0⋅001; or Significance based on 95 % CI.

Multinomial logistic regression.

Adjusted for: race/ethnicity, region, total number of adults living in home, total number of children living in home, frequency of eating out at restaurants, food assistant criterion, FV shopping behaviours (as continuous variables) in only those who are food insecure.

For every one time per month: 0 = Never, 0⋅5 = Less than once a month, 1⋅5 = 1–2 times per month, 4 = 1 time per week, 8 = 2+ times per week.

Among those whose FV intake increased, the frequency of shopping at a food bank/food pantry was 1⋅4 [±2⋅1] times per month, whereas among individuals whose FV intake stayed the same, frequency of shopping at a food bank/food pantry was 1⋅1 [±1⋅8] times per month (*β* = 0⋅1, CI 0⋅0, 0⋅1; *P* = 0⋅05), as shown in Table 2. There was no significant relationship between decreased FV intake and FV shopping behaviour at a food bank/food pantry.

There was also no significant relationship between changes in FV intake and frequency of shopping at a farmers market or food co-op, which represented the least frequented food venue across all FV intake groups.

### Relationship between variables for COVID-19-related concerns and changes in FV consumption

Those reporting concerns about financial stability during COVID-19 had 40 % greater risk of decreased FV intake compared to those not concerned about financial stability (RR (relative risk ratio) 1⋅4; 95 % CI 1⋅0, 2⋅0; *P* = 0⋅03). Additionally, those reporting concerns about employment status changing during COVID-19 had 70 % greater risk of increased FV intake opposed to FV intake staying the same and compared to those not concerned about change of employment status (RR 1⋅7; 95 % CI 1⋅3, 2⋅3; *P* < 0⋅001) (Table 2).

Those reporting concerns about food availability during COVID-19 had 50 % greater risk of increased FV intake opposed to FV intake staying the same and not being concerned for food availability (RR 1⋅5; 95 % CI 1⋅1, 2⋅0; *P* = 0⋅01). On the other hand, those reporting concerns about food availability during COVID-19 had 70 % greater risk of decreased FV intake opposed to FV intake staying the same and not being concerned for food availability (RR 1⋅7; 95 % CI 1⋅3, 2⋅2; *P* < 0⋅001).

Those reporting concerns about food affordability during COVID-19 had 40 % greater risk of increased FV intake opposed to FV intake staying the same and not being concerned for food affordability (RR 1⋅4; 95 % CI 1⋅1, 1⋅8; *P* = 0⋅02). On the other hand, those reporting concerns about food affordability during COVID-19 had 50 % greater risk of decreased FV intake as opposed to FV intake staying the same and not being concerned for food affordability (RR 1⋅5; 95 % CI 1⋅1, 2⋅0 *P* = 0⋅003). Both decreased and increased FV intake were significantly associated with concerns of food affordability during COVID-19.

## Discussion

Our study adds to the growing body of literature to understand how the pandemic impacted dietary behaviours, specifically FV intake in low-income food insecure households. Overall, we found that among households at risk for food insecurity, there were changes in FV intake during this initial phase of the pandemic such that the intake increased or decreased across a majority of the participants, and these changes were associated with several social determinants of health. These results concur with other recent studies that have demonstrated increases in consumption of food products other than produce and changes of food intake patterns during COVID-19^(20)^. A previous study showed lower income was associated with higher boredom- or anxiety-related consumption of foods, disrupted mealtime schedules, decreased purchase of fresh produce and unhealthy changes in overall food consumption^(10)^. Along with behavioural changes, emotional responses arose, which included fear of lack of food as well as feelings of stress and uncertainty. Consumers reduced FV shopping trips to limit the risk of exposure to COVID-19^(21)^. A rise in emotional stress was positively associated with the overconsumption of processed meats and non-perishable items, including processed foods and savoury snacks^(22)^. Panic buying during the crisis included increased non-perishable foods with longer shelf lives and decreased fresh FV^(23)^. The prevalence of food insufficiency, meaning a household did not have enough food to eat within the past week, rose to 13⋅4 % as of December 2020^(3)^. The impact of COVID-19 on the food supply chain and mobility restrictions to purchase fresh produce led to changes in food and dietary behaviours, unsold agriculture products and increased food loss and waste^(21)^. Our study adds to this current body of sparse literature on how the initial phase of the pandemic impacted FV consumption behaviours among food insecure households with children.

Our study demonstrated that respondents who shopped for groceries at small local stores/corner stores reported significantly decreased FV intake; conversely, those who shopped at a food bank/food pantry had significant increases in FV intake. These results underscore the importance of alternative FV access avenues such as food pantries during times of natural disasters to maintain diet quality.

Latest research examined the effects of the pandemic such as COVID-19 fear, social conditions and influences on health and nutrition^(24)^. The pandemic affected individual's daily routines regarding where and how they shopped for produce. Also, fear of COVID-19 positively affected nutritional habits by consuming less unhealthy foods^(24)^. Specifically, individuals consumed more fruit, vitamins and yogurt during this time and less of fast food, snacks, desserts, cigarettes and packaged foods^(24)^. These results aligned with our study where 27 % of the respondents reported increasing their FV intake during that time, even while they reported being food insecure. The present study had a relatively low response rate, while typically the response rate is much higher in this same population, but the findings were sufficient to allow for a pivot in the programme to capture immediate needs of these families which were already utilising the programme prior to the pandemic.

Finally, our results highlight the vulnerability of food insecure households to food availability and affordability. These results concur with other recent studies that evaluated the prevalence of food insecurity associated with affordable, high quality foods and FV intake^(25)^. Those who had access to affordable foods and cooked healthy foods were associated with lower odds of food insecurity overall^(25)^. Individuals who were food insecure were less likely to consume fruits and vegetables^(25)^. Similar to what was identified in our study, other studies demonstrated the impact of the food environment. Individuals using a small local corner store for food purchase may be less likely to find an assortment of healthy foods and thus more inclined to purchasing processed, unhealthy food items with longer shelf life^(26)^. The relationship between food insecurity and FV consumption and shopping behaviours stipulates a need for further interventions to address food insecurity's effects on FV intake. These findings, along with those in our study highlight the disruption of both the food environment, and FV shopping behaviours during the initial phase of the pandemic which in turn impacted produce intake during this time.

### Strengths and limitations

A limitation of the present study was the use of self-reported dietary intake data and the relatively low response rate of 10⋅7 % from a sub-sample of participating families in Brighter Bites in the 2019–2020 school year. However, the intent of this rapid survey was to obtain information on the immediate needs of families during this time to allow organisations like Brighter Bites to pivot services, which was successfully done^(27)^. Selection bias may have occurred due to the electronic nature of the survey including only those who could access this survey and a small proportion of families who participated. However, this was the most effective way to conduct the survey during the pandemic. These survey data provided meaningful insights into context and possible influences on dietary behaviour that have not been studied under the COVID-19 pandemic circumstances in five different regions in the US. However, sample size differed across regions with regards to the proportion of families enrolled in Brighter Bites. Additionally, this sample may not be generalisable to the population across the regions; those who responded may be individuals who needed the most help. In previous studies with a larger sample size, generalisability is high since the Brighter Bites population represents diverse, low-income groups from different regions in the US^(28)^. However, the participants, primarily Hispanic who also qualified for school lunch assistance programme, reflect a sample of convenience, limited to the specific school-based programme, Brighter Bites, and in limited areas of the US; therefore, the study has limited power based on the sample size.

The survey used a well-developed two-item tool to assess and distinguish food insecurity through affirmative responses to at least one of the two questions from the HFSS (Household Food Security Survey)^(17)^. However, the data were self-reported which could result in social desirability bias. Furthermore, modifying the validated questions in the survey to consider changes in FV intake due to the pandemic may have been interpreted in different ways, leading to measurement error. Finally, the cross-sectional nature of this study prevents true causal inferences from being made.

### Implications for research and practice

These results contribute to the literature exploring how unexpected, large-scale pandemic events may impact FV consumption among food insecure families with children. To our knowledge, the present study examined possible influences on dietary behaviour that had not been studied under the COVID-19 pandemic circumstances. In addition, these findings enhance the current literature regarding the dynamic and complex influences on FV consumption. More effective core infrastructure investments are needed to build capacity to diminish the negative impact of natural disasters such as the COVID-19 pandemic on the population's health.

A global approach is needed to decrease food insecurity, a major concern of this pandemic, due to its negative impact on the population's overall health.

## References

[1] World Health Organization (2020) WHO Director-General's Opening Remarks at the Media Briefing on COVID-19-11 March 2020. New York, USA: World Health Organization.

[2] Wolfson JA & Leung CW (2020) Food insecurity and COVID-19: disparities in early effects for US adults. Nutrients 12. doi:10.3390/nu12061648. PMC7352694 32498323

[3] Economic Research Service, U.S. Department of Agriculture. *Food and Consumers*. https://www.ers.usda.gov/covid-19/food-and-consumers/?cpid=email. Updated 2022.

[4] USDA Economic Research Service. *Frequency of Food Insecurity*. https://www.ers.usda.gov/topics/food-nutrition-assistance/food-security-in-the-us/frequency-of-food-insecurity/. Updated 2020.

[5] Drennen CR, Coleman SM, Ettinger de Cuba S, (2019) Food insecurity, health, and development in children under age four years. Pediatrics 144. doi:10.1542/peds.2019-0824. PMC7599443 31501233

[6] Andersen SA (1990) Core indicators of nutritional state for difficult to sample populations. J Nutr 120, 1555. 10.1093/jn/120.suppl_11.1555 2243305

[7] Hales L, Coleman-Jensen A. *Food Insecurity for Households with Children Rose in 2020, Disrupting Decade-Long Decline*. https://www.ers.usda.gov/amber-waves/2022/february/food-insecurity-for-households-with-children-rose-in-2020-disrupting-decade-long-decline/. Updated 2022.

[8] Araújo M, Mendonça R, Lopes Filho JD, (2018) Association between food insecurity and food intake. Nutrition 54, 54–59. doi:10.1016/j.nut.2018.02.023. 29775834

[9] Richards TJ & Rickard B (2020) COVID-19 impact on fruit and vegetable markets. Can J Agric Econ 68, 189–194. doi:10.1111/cjag.12231.

[10] Deschasaux-Tanguy M, Druesne-Pecollo N, Esseddik Y, (2021) Diet and physical activity during the coronavirus disease 2019 (COVID-19) lockdown (March-May 2020): results from the French NutriNet-santé cohort study. Am J Clin Nutr 113, 924–938. doi:10.1093/ajcn/nqaa336. 33675635 PMC7989637

[11] Sharma SV, Markham C, Chow J, (2016) Evaluating a school-based fruit and vegetable co-op in low-income children: a quasi-experimental study. Prev Med 91, 8–17. doi:10.1016/j.ypmed.2016.07.022. 27471022

[12] Marshall AN, Markham C, Ranjit N, (2020) Long-term impact of a school-based nutrition intervention on home nutrition environment and family fruit and vegetable intake: a two-year follow-up study. Prev Med Rep 20, 101247. doi:10.1016/j.pmedr.2020.101247. 33304772 PMC7710647

[13] Hunsberger M, O'Malley J, Block T, (2015) Relative validation of block kids food screener for dietary assessment in children and adolescents. Matern Child Nutr 11, 260–270. doi:10.1111/j.1740-8709.2012.00446.x. 23006452 PMC6860172

[14] Blanck HM, Thompson OM, Nebeling L, (2011) Improving fruit and vegetable consumption: use of farm-to-consumer venues among US adults. Prev Chronic Dis 8, A49. 21324263 PMC3073441

[15] *RTI Surveyed 1,000 Americans about Awareness, Perceptions of COVID-19*. RTI International Website. https://www.rti.org/focus-area/coronavirus-united-states-survey. Updated 2020.

[16] Ding D, Sallis JF, Norman GJ, (2012) Community food environment, home food environment, and fruit and vegetable intake of children and adolescents. J Nutr Educ Behav 44, 634–638. doi:10.1016/j.jneb.2010.07.003. 21531177

[17] Hager ER, Quigg AM, Black MM, (2010) Development and validity of a 2-item screen to identify families at risk for food insecurity. Pediatrics 126, 26. doi:10.1542/peds.2009-3146. 20595453

[18] Evans D, Chaix B, Lobbedez T, (2012) Combining directed acyclic graphs and the change-in-estimate procedure as a novel approach to adjustment-variable selection in epidemiology. BMC Med Res Methodol 12, 156–156. doi:10.1186/1471-2288-12-156. 23058038 PMC3570444

[19] Lee PH (2014) Is a cutoff of 10% appropriate for the change-in-estimate criterion of confounder identification? J Epidemiol 24, 161–167. doi:N/JST.JSTAGE/jea/JE20130062[pii]. 24317343 10.2188/jea.JE20130062 PMC3983286

[20] Górnicka M, Drywień ME, Zielinska MA, (2020) Dietary and lifestyle changes during COVID-19 and the subsequent lockdowns among Polish adults: a cross-sectional online survey PLifeCOVID-19 study. Nutrients 12. doi:10.3390/nu12082324. PMC7468840 32756458

[21] Hassen TB, Bilali HE & Allahyari MS (2020) Impact of COVID-19 on food behavior and consumption in Qatar. Sustainability 12, 1–18.35136666

[22] Sadler JR, Thapaliya G, Jansen E, (2021) COVID-19 stress and food intake: protective and risk factors for stress-related palatable food intake in U.S. adults. Nutrients 13. doi:10.3390/nu13030901. PMC8000206 33802066

[23] Baker SR, Farrokhnia RA, Meyer S, (2020) *How Does Household Spending Respond to an Epidemic? Consumption during the 2020 COVID-19 Pandemic*. NBER Working Papers. https://ideas.repec.org/p/nbr/nberwo/26949.html (accessed April 16, 2021).

[24] Aksoy NC, Kabadayi ET & Alan AK (2021) An unintended consequence of COVID-19: healthy nutrition. Appetite 166, 105430. doi:10.1016/j.appet.2021.105430. 34089803 PMC9756095

[25] Horning ML, Alver B, Porter L, (2021) Food insecurity, food-related characteristics and behaviors, and fruit and vegetable intake in mobile market customers. Appetite, 105466. doi:10.1016/j.appet.2021.105466. 34139297 PMC8445326

[26] HealthValueHub. https://www.healthcarevaluehub.org/advocate-resources/publications/social-determinants-health-food-insecurity-united-states (accessed June 16, 2021).

[27] Haidar A, Khoei A, Alex SE, (2021) Community-academic partnerships to promote health literacy and address social needs among low-income families during COVID-19. J Nutr Educ Behav 53, 75–78. doi:10.1016/j.jneb.2020.10.003. 33187874 PMC7561286

[28] Crulcich S (2019) The Impact of Food Security Status on the Food Shopping Behavior and Patterns of 2018 Brighter Bites Participants at Baseline. UT School of Public Health Dissertations.

